# Therapeutic Drug Monitoring of Elexacaftor, Tezacaftor, and Ivacaftor in Adult People with Cystic Fibrosis

**DOI:** 10.3390/jpm14101065

**Published:** 2024-10-17

**Authors:** Susanne Naehrig, Christina Shad, Magdalena Breuling, Melanie Goetschke, Katharina Habler, Sarah Sieber, Johanna Kastenberger, Alexandra Katharina Kunzelmann, Olaf Sommerburg, Uwe Liebchen, Juergen Behr, Michael Vogeser, Michael Paal

**Affiliations:** 1 Cystic Fibrosis Center for Adults, Department of Medicine V, LMU University Hospital, LMU Munich, Comprehensive Pneumology Center, Member of the German Center for Lung Research (DZL), 80336 Munich, Germany; 2 Institute of Laboratory Medicine, LMU University Hospital, LMU Munich, 813777 Munich, Germany; 3 STAT-UP Statistical Consulting & Data Science GmbH, 80333 Munich, Germany; 4 Department of Anesthesiology, LMU University Hospital, LMU Munich, 81377 Munich, Germany; 5 Division of Pediatric Pulmonology, Allergy and Cystic Fibrosis Center, Department of Pediatrics, University Hospital Heidelberg, Member of the German Center for Lung Research (DZL), 69120 Heidelberg, Germany; olaf.sommerburg@med.uni-heidelberg.de; 6 Department of Medicine V, LMU University Hospital, LMU Munich, Comprehensive Pneumology Center, Member of the German Center for Lung Research (DZL), 81377 Munich, Germany; juergen.behr@med.uni-muenchen.de

**Keywords:** therapeutic drug monitoring, elexacaftor, tezacaftor, ivacaftor, cystic fibrosis, serum concentrations

## Abstract

Background/Objectives: Elexacaftor, tezacaftor, and ivacaftor (ETI) have significantly improved lung function in people with cystic fibrosis (pwCF). Despite exceptional improvements in most cases, treatment-related inter-subject variability and drug–drug interactions that complicate modulator therapy have been reported. Methods: This retrospective analysis presents data on the serum concentration of ETI in our pwCF with full or reduced dosage from August 2021 to December 2023 via routine therapeutic drug monitoring (TDM). The data were compared with the maximum drug concentrations (Cmax) from the pharmaceutical company’s summary of product characteristics. Results: A total of 786 blood samples from 155 pwCF (41% female, 59% male) were analyzed. The examinations were divided into four groups: full dose within the given tmax (38.5% of all measurements), full dose outside the tmax (29%), reduced dose within the tmax (19.2%), and reduced dose outside the tmax (13.2%). In pwCF receiving the full dose and blood taken within the tmax, 45.3% of serum concentrations of elexacaftor, 51.1% of serum concentrations of ivacaftor, and 8.9% of serum concentrations of tezacaftor were found to be above the Cmax, respectively. For those on reduced doses within the tmax, 24.5% had a serum concentration of elexacaftor, 23.2% had a serum concentration of ivacaftor, and 2.5% had a serum concentration of tezacaftor above the Cmax, respectively. Conclusions: Many pwCF under ETI therapy have Cmax values for elexacaftor and ivacaftor above the recommended range, even on reduced doses or before the tmax was reached. This highlights the value of a TDM program. Further pharmacokinetic studies are necessary.

## 1. Introduction

Cystic fibrosis (CF) is the most common autosomal-recessive disease in the Caucasian population. The genetic defect affects the gene encoding for the cystic fibrosis transmembrane conductance regulator (CFTR) protein, which is responsible for chloride and bicarbonate channels on the apical surface of epithelial cells [1]. CF affects multiple organs, with acute and chronic lung infections and progressive lung disease being the leading causes of morbidity and mortality. Consequently, antibiotic and anti-inflammatory therapies were the cornerstones of CF therapy [2]. More recently, specific target-based therapies have been developed. CFTR modulators are small molecules that bind to the defective CFTR protein [3]. In August 2020, a triple combination of CFTR modulators, containing elexacaftor, tezacaftor, and ivacaftor (ETI), was licensed in the EU to treat people living with cystic fibrosis (pwCF) with at least one F508del mutation. In phase III studies, ETI showed significant improvement in the percentage of predicted forced expiratory volume in the first second (ppFEV1) and quality of life, as well as a significant decrease in sweat chloride concentration and reduction in pulmonary exacerbations [4,5]. Those effects were sustained over 144 weeks [6]. Real-life studies and registry data confirmed those impressive improvements [7,8,9,10]. Approximately 85–90% of pwCF can now be treated with this highly effective therapy.

Despite exceptional improvements in most cases, treatment-related inter-subject variability has been reported. In heterozygous pwCF, the change in ppFEV1 after 24 weeks of treatment with ETI was from −10% or less up to plus 30% or more [4]. In homozygous pwCF, the individual response of ppFEV1 with ETI was between −2.5% and >+20% [5]. Neither study revealed any explanations for this wide range of individual response. It was discussed that the same dosage of ETI might result in differences in drug exposure and, thereby, variation in treatment response [11]. Van der Meer R et al. showed that the pancreatic status (pancreatic-sufficient or pancreatic-insufficient) did not significantly change the pharmacokinetic parameters of ivacaftor [12].

All three drug components are metabolized in the liver, primarily through cytochrome P450 3A (CYP3A), including both isoforms CYP3A4 and CYP3A5. Drug–drug interactions are possible due to primary metabolism via the CYP enzyme system. Due to extensive co-medication with potent CYP3A inhibitors, e.g., macrolide antibiotics, azole antifungals, or strong CYP3A inducers, e.g., glucocorticoids or rifampicin, pwCF have a considerable risk of relevant drug interactions, resulting in lower or higher serum concentrations of ETI, respectively [3]. When these drugs are used simultaneously, the CFTR modulator dose should be reduced according to the prescribing information [13]. Jansen et al. showed, just recently, that the concomitant application of itraconazole or posaconazol and an adapted ETI dose led to subtherapeutic azole concentrations [14]. A review by Purkayastha and colleagues identified 86 potential drug interactions via the CYP family, while evidence was available in only 13 published cases [15]. Furthermore, interactions with transport proteins like OATP1B1 and P-glycoprotein have been described previously [16]. Rose et al. showed that there is a pharmacokinetic variability of ETI in pwCF on standard or reduced doses, but after dose equivalence normalization, the differences were non-significant. They suggested that dose reduction might be a viable strategy to manage side effects, although further studies are needed [17].

The aforementioned factors may lead to unpredictable individual drug exposure. Our Institute of Laboratory Medicine established an isotope dilution LC-MS/MS method to measure elexacaftor, tezacaftor, and ivacaftor [18] in pwCF serum for therapeutic drug monitoring (TDM) to optimize care. This retrospective analysis presents comprehensive data over 2.5 years on serum concentrations of ETI in pwCF with full or reduced doses. Additionally, the effects of age, gender, BMI, ppFEV1, mutation status, and pancreatic status on serum concentrations of ETI were examined retrospectively.

## 2. Materials and Methods

### 2.1. Study Subjects and Design

Observational data from adult pwCF were generated during routine outpatient clinic visits at the CF Center for Adults at the LMU University Hospital, Munich, with an approximate three months interval between August 2021 and December 2023. Patients were included in this analysis if they had taken ETI in the same dosage for at least four weeks to be in a steady state and if they had given written informed consent. We excluded measurements of female pwCF during pregnancy as well as any measurement more than 12 h after administration of ETI.

### 2.2. Data and Bio-Sample Collection

#### 2.2.1. Blood Sample Collection

PwCF consistently followed their medication regimen and attended daytime clinic appointments. Serum blood samples were routinely collected from adult patients during outpatient clinic visits in accordance with standard clinical practice, without adherence to a predetermined schedule or timing protocol. The serum concentrations of ETI were assessed using residual blood samples obtained during routine procedures. They were instructed to take ETI in the morning and IVA at night with a fat-containing meal, approximately 12 h apart. Compliance with therapeutic regimens was assessed during each study visit, with any deviations carefully documented. Patients were asked about the time of the last medication intake, and the answer was documented as well as the time when blood was taken. For the analysis, the time interval between the previous medication intake and blood collection was determined and rounded up or down to the nearest 15 min mark.

#### 2.2.2. Quantification of ETI and Dose

ETI was quantified according to isotope dilution liquid chromatography tandem mass spectrometry (ID-LC-MS/MS), regarded as the gold standard for therapeutic drug monitoring (TDM). We utilized a laboratory-developed ID-LC-MS/MS test, as described by Habler K et al. 2021, designed to simultaneously quantify all three components in human serum [18]. After centrifugation, patient sera were stably stored in polypropylene tubes at −20 °C up to 14 days until routine LC-MS/MS analysis for ETI. Briefly, following sample cleanup by protein precipitation, the analytes were separated using a two-dimensional chromatography and detected in positive electrospray ionization mode in multiple reaction monitoring on a Waters Xevo TQ-XS tandem mass spectrometer (Waters, Milford, MA, USA).

Each sample was tested for all three components. Concentrations were compared with the maximum drug concentrations (Cmax) from the summary of product characteristics provided by the pharmaceutical company. As per the European Medicines Agency, the reported Cmax ± SD concentrations after administration are 9.15 ± 2.09 µg/mL for elexacaftor, 7.67 ± 1.68 µg/mL for tezacaftor, and 1.24 ± 0.34 µg/mL for ivacaftor. The median (range) absorption time to reach Cmax (tmax) is 6 h (4–12 h), 3 h (2–4 h), and 4 h (3–6 h), respectively [19]. So, each component was evaluated within its specific tmax and Cmax ranges.

The statistical analysis was conducted retrospectively. However, as serum values were available approximately two weeks after each patient’s visit, in some patients, the dose was reduced in consultation with the patient, e.g., if side effects were identified (depressed mood, diarrhea, elevated bilirubin) and/or when ETI concentrations exceeded the maximum drug concentrations given in the product information. The reason for the dose reduction and subsequent adjustment were documented in the patient‘s chart.

According to the product information, the dosing recommendation for the regular dose of ETI in adults is elexacaftor/tezacaftor/ivacaftor 100/50/75 mg, two tablets in the morning, and ivacaftor 150 mg, one tablet in the evening. The most common dose reduction was elexacaftor/tezacaftor/ivacaftor 100/50/75 mg, one tablet in the morning and a maintained evening dose of ivacaftor 150 mg, one tablet. Other reduced doses included no ivacaftor in the evening or elexacaftor/tezacaftor/ivacaftor 100/50/75 mg, one tablet in the morning and ivacaftor 75 mg, one tablet in the evening. If the dose had to be reduced because of concomitant azole therapy, the dose reduction followed the product information (two tablets elexacaftor/tezacaftor/ivacaftor 100/50/75 mg in the morning on day 1, 4, and 7 and no ivacaftor in the evening).

#### 2.2.3. Respiratory Status

Pulmonary function was routinely assessed at each outpatient visit. Spirometry (MasterScreen, Vyaire) was conducted by trained personnel according to standardized procedures outlined in the ATS/ERS guidelines [20]. Global Lung Initiative Equations determined ppFEV1 [21].

#### 2.2.4. Other Study Parameters

All other clinical or laboratory parameters were extracted once from our laboratory system or collected by chart review. BMI was calculated by dividing body weight (kg) by the square of height (m).

### 2.3. Statistical Analysis

The retrospective statistical analysis for this study was performed using R version 4.3.2. To calculate the proportions of pwCF below, within, and above recommended serum levels, the number of data points per category was calculated and subsequently divided by the number of total data points.

Baseline characteristics, such as BMI, age, and initial ppFEV1 value, are represented by the respective median value and the range from minimum to maximum values. The serum level (µg/mL) by reduced and full doses within and outside the given tmax is described by the median value and interquartile range (IQR).

To determine the effect of age, BMI, and ppFEV1 on serum concentrations and to take repeated measurements into account, linear mixed models were calculated using the subject ID as random intercept. The models were run separately for reduced and full doses and active substances. For better interpretation, age and ppFEV1 were scaled by a factor of five. The *p*-values of the model effects were not corrected for multiple testing and should be interpreted in an exploratory sense.

Scatterplots are used to visualize the values of certain variables over time. A line is fitted to the data points to further demonstrate trends in the behavior of the variables. In cases where the data points fluctuate greatly, locally estimated scatterplot smoothing (LOESS) is utilized. LOESS produces a smooth curve that fits the data points in a scatterplot. These curves are fitted with a 95% confidence interval.

### 2.4. Ethical Statement

This study was in accordance with the guidelines of the Declaration of Helsinki and approved by the Institutional Review Board of the Ludwig-Maximilians-Universität (LMU) Munich (protocol number 20-0999); approval was given on 12 January 2021. 

## 3. Results

### 3.1. Baseline Demographics

Between August 2021 and December 2023, 155 pwCF (41% female, 59% male) with up to nine measurement dates were included in our analysis. The median age was 38 years (range 21–68 years). The median ppFEV1 was 76%. The median BMI was 22.5 kg/m^2^. A total of 63.2% were F508del homozygous. For all baseline characteristics, please see Table 1. Table S1 shows the number of pwCF with data for each timepoint available.

### 3.2. Blood Samples

**Figure 1:**
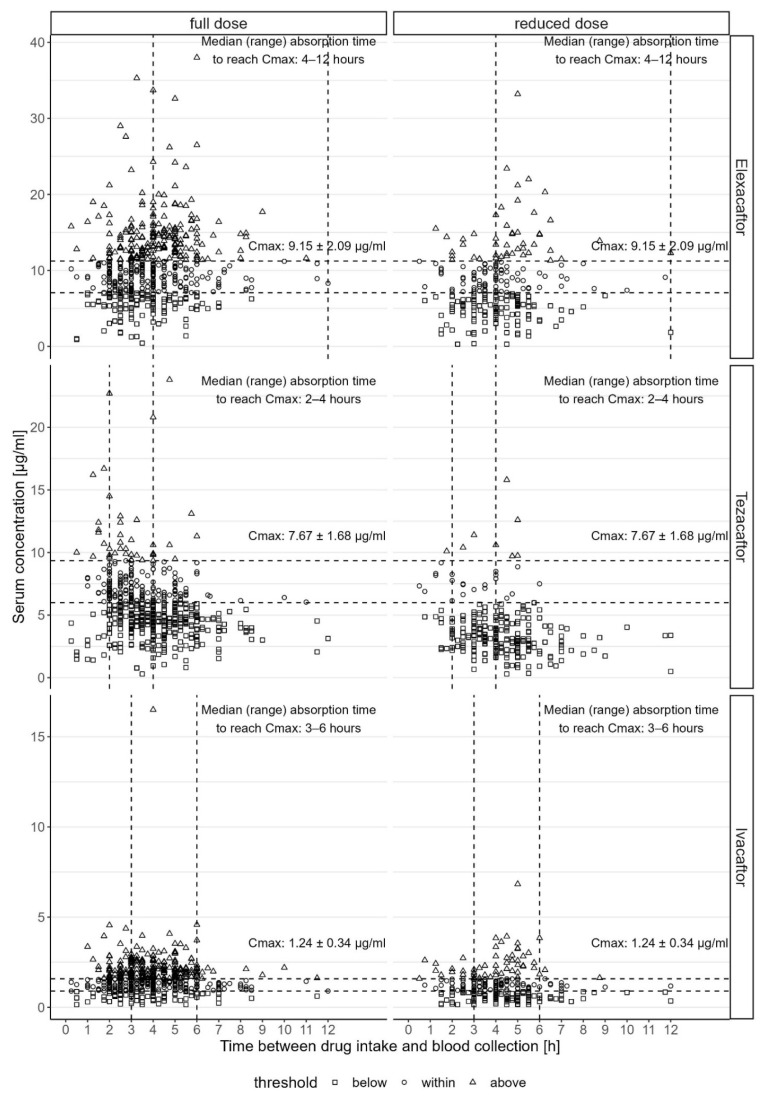
Scatterplots of all serum concentrations for elexacaftor, tezacaftor, and ivacaftor with full vs. reduced doses. Horizontal lines display the Cmax ± SD, and vertical lines display the median absorption timeframe (tmax) to reach Cmax for each active substance according to the summary of product information (European Medicines Agency 2020).

A total of 786 blood samples were taken from 155 pwCF. A total of 26 measurements of female pwCF were excluded during pregnancy as well as *n* = 136 blood samples with examinations more than 12 h after administration of ETI. In each blood sample, the serum concentrations of elexacaftor, tezacaftor, and ivacaftor were measured. A total of 127 pwCF took the full dose of ETI at the start of the analysis, and 28 pwCF started on a reduced dose. The reasons for a reduced dose at the beginning were increased liver enzymes (*n* = 6), gastrointestinal side effects (*n* = 6), patient’s wish out of fear for side effects (*n* = 4), headache (*n* = 3), depressed mood (*n* = 3), liver cirrhosis according to product information (*n* = 2), highly elevated serum values for elexacaftor before the start of the analysis (*n* = 2), and comedication (*n* = 2). With regard to the different timeframes for Cmax for each of the three compounds given in the product information and because not all pwCF took the full recommended dose, the measurements were divided into four groups: samples after full dose within the recommended tmax (38.5% of all measurements), full dose outside the tmax (29% of all measurements), reduced dose within the tmax (19.2% of all measurements), and reduced dose outside the tmax (13.2% of all measurements). Table 2 shows the number of examinations for each group and compound and the corresponding mean Cmax ± SD value. Also given are the percentages of values below, within, or above the Cmax ranges given in the product information. Figure 1 shows the scatterplots of all values for elexacaftor, tezacaftor, and ivacaftor. During the entire data analysis period, the dose was reduced in six pwCF due to comedication with itraconazole or posaconazole, which affected 16 measurements. Of those, *n* = 1 measurements of elexacaftor showed a value above Cmax, and *n* = 10 measurements of ivacaftor showed a value above Cmax.

### 3.3. Impact of Other Variables on Serum Concentrations

#### 3.3.1. Age, BMI, and ppFEV1

In pwCF receiving the full ETI dose, no effect of age on serum concentrations was observed. Concerning BMI, there was a weak negative effect on tezacaftor (−0.12; *p*-value below 0.05 but not corrected for multiple testing) with the full dose and a weak positive effect on elexacaftor with a reduced dose (0.28). There was a weak negative effect for ppFEV1 on elexacaftor in pwCF on a reduced dose of −0.15. This means that an increase in ppFEV1 by 5% correlates with a decrease in serum level by 0.15. Table 3 provides a summary of the linear mixed model effects between serum concentrations and age, BMI, and ppFEV1. The scatterplots are in the Figures S1–S3.

#### 3.3.2. Gender and Mutation Status

Gender did not influence serum concentrations, neither on the full nor reduced doses (see Figure S4).

**Figure 2:**
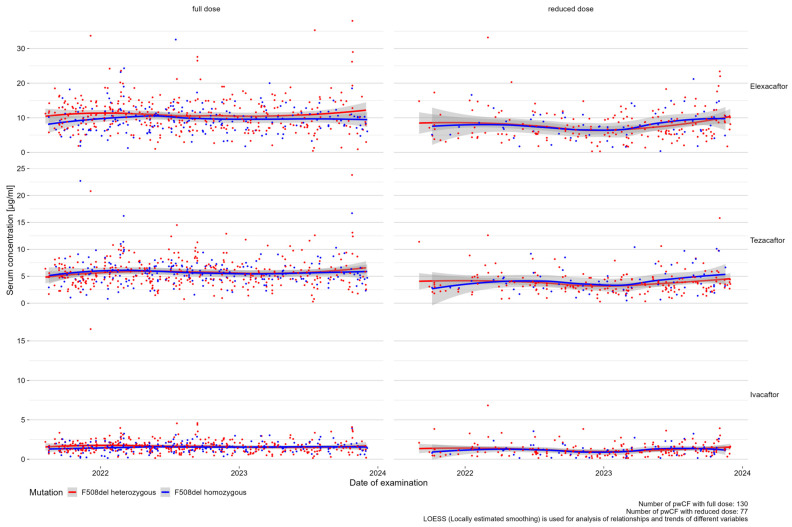
Effects between mutation status and serum concentration of elexacaftor, tezacaftor, and ivacaftor.

Concerning mutation status, serum values for elexacaftor from heterozygous pwCF on the full dose seemed to be slightly higher than from F508del homozygous pwCF (see Figure 2). This effect was not observed for tezacaftor and ivacaftor or for measurements from pwCF on a reduced dose.

#### 3.3.3. Pancreas Status

**Figure 3:**
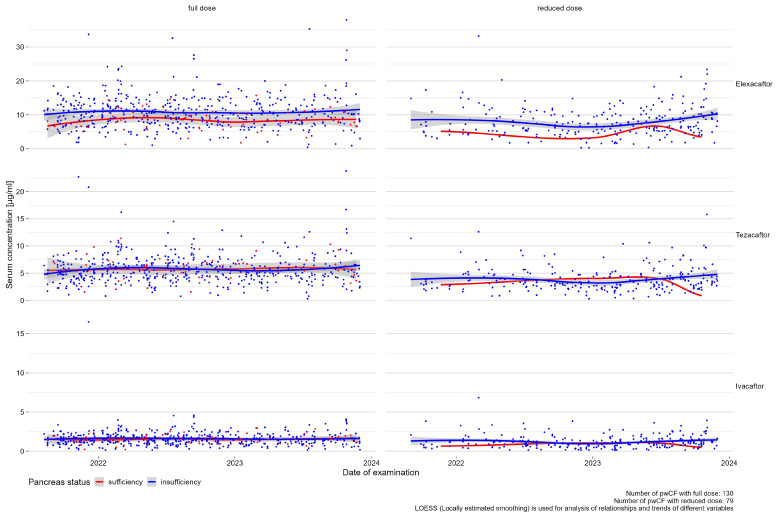
Effects between pancreatic status and serum concentrations of ETI.

Serum concentrations of elexacaftor from pwCF on the full dose seemed higher in insufficient pancreatic patients. For tezacaftor or ivacaftor, this effect was not observed (see Figure 3).

## 4. Discussion

Our single-center cohort study presents a retrospective analysis of real-world data on serum concentrations of ETI in *n* = 155 adult pwCF over 29 months. Measurements of serum concentrations of ETI have been part of our routine TDM for pwCF on modulator therapy since August 2021. At that timepoint, most of the eligible pwCF had already started with ETI several months ago and, therefore, were in a steady state. To our knowledge, this is the most extensive dataset published to date, including *n* = 786 blood samples with measurements of serum concentrations of ETI in adult pwCF.

The results showed that, when blood was drawn within the median range absorption time, of the pwCF receiving the full dose of ETI, 45.3% had serum concentrations of elexacaftor above the threshold mentioned in the product information, 51.1% had serum concentrations of ivacaftor above the threshold, and 8.9% had serum concentrations of tezacaftor above the threshold. Even 24.5% of measurements from pwCF receiving a reduced dose had elexacaftor concentrations above the Cmax values. Concerning ivacaftor, 23.2% of measurements on a reduced dose were elevated. Ten measurements in pwCF on a reduced dose due to comedication with itraconazole or posaconazole revealed elevated values for ivacaftor. Remarkably, for tezacaftor on the full dose, more than half of all measurements within the tmax were below the Cmax concentrations given in the manufacturer’s summary of product characteristics (52.2% of measurements). Interestingly, a significant portion of measurements in pwCF on the full dose but taken before the tmax had elevated serum concentrations. Remarkably, even in patients on reduced doses, elevated serum concentrations were observed for both elexacaftor and ivacaftor. Exploring the evolution of serum concentrations over time in pwCF would provide valuable insights.

Only limited pharmacokinetic data about ETI have been published so far. In the summary of product characteristics, values for Cmax and AUC are given for each of the three compounds [19]. In that document, it is stated (Section 5.2) that the pharmacokinetics of elexacaftor, tezacaftor, and ivacaftor are similar between healthy adult subjects and pwCF. Interestingly, in a phase I study, the AUC of elexacaftor was increased 1.25-fold in non-CF subjects with moderate hepatic impairment compared to matched healthy subjects [22]. In a phase II study, a dose-dependent effect was shown for ppFEV1, sweat chloride, and the Cystic Fibrosis Questionnaire-Revised (CFQ-R) respiratory domain score in 51 patients heterozygous for F508del. Based on this small cohort, the highest dose was chosen for all following studies and all mutations, despite clinical effectiveness with lower doses [23]. In a phase III study in children aged 6–11 years, the AUC values from two previous phase III studies in adults [4,5], but not published in the respective phase III studies] were published for the first time in a graph as a comparison to the AUC values of the pediatric study population [24]. The figure showed a large spread of AUC values for all three components. Vonk et al. stated, just recently, that good therapeutic reference ranges are lacking [25]. The study by Rose et al. involving 15 pwCF on CFTR modulator therapy found that, despite reducing the medication in eight cases, there was no significant evidence of a difference in the ETI plasma concentration (Cmin) compared to the standard dose. Most of the concentrations measured were expected to elicit a clinical response to ETI therapy, suggesting that a reduction in dosage could be a potential approach [17].

The registration documents provide reference ranges for AUC, Cmin/Cmax [26], and Cmax ranges as well as AUCs reported by the manufacturer [19]. In 2022, Ryan et al. provided concentration–time curves of ETI of *n* = 6 pwCF on modulator therapy [27]. The measured values were compared with the Cmin and Cmax concentrations reported by the manufacturer. Interestingly, the concentrations observed in our investigation significantly exceed those observed by Ryan and colleagues (2022). Our findings have prompted several thought-provoking questions: What is the clinical significance of nearly 50% of pwCF and ETI therapy having Cmax values for elexacaftor and ivacaftor above the range specified in the product information? Conversely, how can we account for 52% of tezacaftor concentrations falling below the given Cmax range, potentially indicating reduced effectiveness? What are the main factors that influence serum concentrations of ETI? Would a reduced dose of these modulators have the same clinical efficacy in special patient groups? Are there any risks for patients with elevated Cmax values? Similar questions have been raised by van der Meer et al. concerning the dosing of ivacaftor [11].

We identified a weak positive effect between BMI and elexacaftor for pwCF and a reduced dose of ETI. We also found statistically significant negative effects between BMI and Cmax values for tezacaftor in pwCF on the full dose of ETI as well as between ppFEV1 and elexacaftor on a reduced dose, as pwCF and lower ppFEV1 values had higher Cmax values. However, these data should be interpreted with caution due to the retrospective nature of this study. It might be interesting to see if a pharmacokinetic study can confirm these weak effects. For ivacaftor, in vitro data showed that ivacaftor can destabilize corrected F508del CFTR [28,29]. While this effect, if present, does not prevent a clinical benefit from CFTR modulator therapy with current dosing, it may be limiting these agents’ potential. There was a trend for higher serum concentrations for elexacaftor (full dose) in pwCF and only one F508del mutation. However, as confidence intervals overlap, this effect is considered insignificant. Correlation between pancreas status and serum concentrations of ETI showed that patients with pancreas insufficiency showed higher elexacaftor concentrations under the full dose of ETI. The bioavailability (AUC) of elexacaftor is increased by 1.9-fold to 2.5-fold when administered with a fatty meal. Ivacaftor’s bioavailability (AUC) increased even more (2.5–5-fold), while fatty food does not affect the exposure of TEZ [19]. It might be possible that pwCF with a pancreas insufficiency taking pancreatic enzyme replacement therapy have better absorption of elexacaftor than pancreatic-sufficient patients. However, for ivacaftor, van der Meer et al. did not find an impact of exocrine pancreatic function or the use of pancreatic enzymes in pwCF on absorption or exposure of ivacaftor concentration [12]. In our subanalyses, we did not find any factors, such as age, BMI, ppFEV1, mutation status, or pancreas status, that appeared to have a significant impact on the serum concentrations of ETI. Further studies are necessary to validate the preliminary results we have obtained.

The ETI dose was reduced in many pwCF, often in response to side effects. After the licensing of ETI, several case reports have been published about the development of mental health disorders after the initiation of ETI treatment [30,31,32]. None of the case reports mentioned the measurements of serum levels of ETI. However, in two reports, the reduction in ETI doses led to improved mental health [33,34]. Another common side effect of treatment with ETI is an increase in liver enzymes, which has previously been noted in phase III studies. Elevations of alanine transaminase or aspartate transaminase more than three-fold or five-fold above the upper limit of normal (ULN) happened in 7% and 4% of F508del homozygous patients [5] and 7.9% and 2.5% of heterozygous patients [4], respectively. However, in real-world settings, transaminase elevations of more than three times the ULN were reported in 11% [35]. In that study, 75% of patients experienced an elevation of 25% or more above baseline. Two studies showed that dose reduction because of either increased liver enzymes [35] or newly developed neuropsychiatric symptoms [33] did not cause impaired efficacy but improved side effects. As a growing number of people with CF gain access to CFTR modulators, it is crucial to be aware of potential adverse events and to optimize patient safety [36].

Our study has several limitations that make interpretation challenging. We present data from a retrospective, single-center study. We did not have a protocol standardizing the timepoints for blood draws from timing ETI, resulting in samples collected both within and outside of the tmax given in the product information. As a result, we had to rely on patient recall to determine the timing of their dose. Secondly, we measured ETI concentrations once per visit instead of AUC measurements, which require multiple measurements in one day. AUC measurements have the advantage of presenting the total amounts of drug exposure, but according to the German Medicines Law, AUC measurements would have required a true clinical study. At the time, we did not have funding for a clinical trial. Third, we measured serum concentrations; however, since the place of action is within cells, intracellular concentrations might provide a better correlation with efficacy. Guimbellot JS et al. 2020 observed a correlation between plasma and cellular ivacaftor concentrations, but cellular concentrations were disproportionally more elevated in patients with higher plasma concentrations [37]. This suggests in vivo accumulation of ivacaftor [38]. The higher cellular concentrations may result in a level of CFTR restoration distinct from what would be expected from plasma concentrations. Finally, we did not evaluate ETI metabolites. Especially, the M23-elexacaftor metabolite seems to play a role in patients without cystic fibrosis but moderate hepatic impairment [22]. Hence, prospective clinical studies are necessary to ultimately address these questions, including studies with AUC measurements and/or Cmin [25] and a correlation with lung function and sweat chloride measurements.

Our retrospective analysis did not intend to show any correlation between serum values, side effects, and efficacy. According to German Medicines Law, any study evaluating efficacy, side effects, or pharmacokinetics is considered a clinical trial, subject to certain restrictions. However, despite the mentioned limitations, our retrospectively analyzed data from real-life pwCF revealed that most of the pwCF who were taking ETI did not have Cmax values within the range recommended in the product information. Our findings indicate a wide variation in how individuals respond to the treatment. Therefore, it may be beneficial to enhance patient care by implementing a TDM program to monitor ETI concentrations in all patients, especially those experiencing side effects, unexpected symptoms, or unusual responses, as well as in specific scenarios such as pregnancy. This approach could be valuable in clinical practice and improve safety and treatment outcomes. We now applied for funding for a clinical study with measurements of Cmin, AUC, ppFEV1, and sweat chloride; so hopefully, we will be able to gain further information about the pharmacokinetics of ETI in pwCF soon.

In the future, the development of a pharmacokinetic/pharmacodynamic model could facilitate the identification of individualized dosing regimens for each patient, employing the Bayesian Dosing principle to achieve “the right dose for the right patient at the right time”. Besides an increase in patient safety, there is also the chance of saving costs if the dose could be reduced in some patients without compromising efficacy.

## 5. Conclusions

A significant percentage of pwCF under ETI therapy have elevated Cmax values for elexacaftor and ivacaftor, even on reduced doses. Therefore, a TDM program is recommended. Further pharmacokinetic studies are necessary to individualize dose adjustments at the patient level and to contribute to excellent therapeutic safety and efficiency. Our findings also highlight the necessity for long-term research to comprehend how high blood concentrations affect patients’ health.

## Figures and Tables

**Table 1:** Baseline characteristics of all pwCF.

Total Number of pwCF, *n*	155
Male, *n* (%)	91 (59)
Female, *n* (%)	64 (41)
Median age, years (range)	38 (21–68)
Median BMI, kg/m^2^ (range)	22.5 (15.7–33.1)
Median ppFEV1, % (range)	76 (27–126)
Mutation status	
delF508 homozygous, *n* (%)	98 (63.2)
delF508 heterozygous, *n* (%)	47 (30.3)
other mutations, *n* (%)	10 (6.5)
Pancreatic insufficiency, *n* (%)	139 (89.7)

Data are presented as *n* (%) or median (range). ppFEV1, percent predicted forced expiratory volume in one second; pwCF, people with cystic fibrosis.

**Table 2:** Overview of examinations and ETI concentrations.

Category ^1^	Active Substance	Number of Measurements (*n*)	Serum Concentrations, µg/mL Median (IQR)	Measurements below C_max_ ^2^ [%]	Measurements within C_max_ ^2^ [%]	Measurements above C_max_ ^2^ [%]
Full dose, within t_max_ (38.5% of all measurements)	Elexacaftor	265	10.7 (7.95; 14.30)	17.7	37.0	45.3
	Tezacaftor	291	5.8 (4.52; 7.25)	52.2	38.8	8.9
	Ivacaftor ^3^	352	1.6 (1.16; 2.08)	12.5	36.4	51.1
Full dose, out of t_max_ (29% of all measurements)	Elexacaftor	266	10.1 (7.37; 12.95)	22.6	39.5	37.9
	Tezacaftor	240	5.3 (4.22; 6.83)	60.6	32.0	7.3
	Ivacaftor	178	1.5 (1.07; 1.98)	14.7	41.3	44.0
Reduced dose, within t_max_ (19.2% of all measurements)	Elexacaftor	151	7.2 (5.12; 11.00)	48.3	27.2	24.5
	Tezacaftor	121	3.6 (2.86; 4.72)	87.6	9.9	2.5
	Ivacaftor	181	1.1 (0.63; 1.55)	39.2	37.6	23.2
Reduced dose, out of t_max_ (13.2% of all measurements)	Elexacaftor	104	7.0 (5.11; 10.20)	50.2	31.0	18.8
	Tezacaftor	134	3.4 (2.43; 4.77)	87.8	8.6	3.5
	Ivacaftor	74	1.1 (0.65; 1.53)	39.2	39.6	21.2

^1^ Categories based on median absorption time range for ETI to reach Cmax according to the summary of product characteristics. Median (range) absorption time to maximum concentration (tmax): elexacaftor, approx. 6 h (4–12 h); tezacaftor, approx. 3 h (2–4 h); ivacaftor, approx. 4 h (3–6 h) (European Medicines Agency 2020). ^2^ Maximum observed drug concentrations (Cmax) from the summary of product characteristics provided by the pharmaceutical company based on median (range) tmax. Cmax ± SD concentrations after administration of ETI: 9.15 ± 2.09 μg/mL (elexacaftor), 7.67 ± 1.68 μg/mL (tezacaftor), 1.24 ± 0.34 μg/mL (ivacaftor) (European medicines Agency 2020). ^3^ One missing value for ivacaftor for the examination of one patient with full dose within tmax. Numbers are rounded to one figure after the decimal.

**Table 3:** Effects of age, BMI, and ppFEV1 on serum concentrations of ETI.

	Full Dose			Reduced Dose		
	ELX	TEZ	IVA	ELX	TEZ	IVA
Age ^1^	ns	ns	ns	ns	ns	ns
BMI ^1^	ns	−0.12	ns	0.28	ns	ns
ppFEV1 ^2^	ns	ns	ns	−0.15	ns	ns

^1^ Number of pwCF with full dose: 131; number of pwCF with reduced dose: 79. ^2^ Number of pwCF with full dose: 131; number of pwCF with reduced dose: 77. Data are linear mixed model fixed effect coefficients. Significance level: *p* < 0.05 (not corrected for multiple testing). ELX, elexacaftor; IVA, ivacaftor; ns, not significant; ppFEV1, percent predicted forced expiratory volume in one second; pwCF, people with cystic fibrosis; TEZ, tezacaftor.

## Data Availability

All data can be presented on request.

## Supplementary Materials

The following supporting information can be downloaded at: https://www.mdpi.com/article/10.3390/jpm14101065/s1, Table S1: Number of pwCF with data for each time point available, Figure S1: Effects between age and serum concentrations of ETI, Figure S2: Effects between BMI and serum levels of ETI; Figure S3: Effects between ppFEV1 and serum levels, Figure S4: Effects between gender and serum levels 4.
