# Supplementary material for: Therapeutic Drug Monitoring of Elexacaftor, Tezacaftor, and Ivacaftor in Adult People with Cystic Fibrosis
Source: J Pers Med. 2024 Oct 17;14(10):1065. doi: 10.3390/jpm14101065 (PMC11508966; doi:10.3390/jpm14101065)
Supplement: Supplementary file 1 [file jpm-14-01065-s001.zip › jpm-3201149-supplementary.pdf]

Article

Therapeutic Drug Monitoring of Elexacaftor, Tezacaftor, and Ivacaftor in Adult People with Cystic Fibrosis

Susanne Naehrig [1,*], Christina Shad [1], Magdalena Breuling [1], Melanie Goetschke [1], Katharina Habler [2], Sarah Sieber [3], Johanna Kastenberger [3], Alexandra Katharina Kunzelmann [4], Olaf Sommerburg [5], Uwe Liebchen [4], Juergen Behr [6], Michael Vogeser [2] and Michael Paal [2]

[1] Cystic Fibrosis Center for Adults, Department of Medicine V, LMU University Hospital, LMU Munich, Comprehensive Pneumology Center, Member of the German Center for Lung Research (DZL); 80336 Munich, Germany
[2] Institute of Laboratory Medicine, LMU University Hospital, LMU Munich, 813777 Munich, Germany
[3] STAT-UP Statistical Consulting & Data Science GmbH, 80333 Munich, Germany
[4] Department of Anesthesiology, LMU University Hospital, LMU Munich, 81377 Munich, Germany
[5] Division of Pediatric Pulmonology, Allergy and Cystic Fibrosis Center, Department of Pediatrics, University Hospital Heidelberg, Member of the German Center for Lung Research (DZL), 69120 Heidelberg, Germany; olaf.sommerburg@med.uni-heidelberg.de
[6] Department of Medicine V, LMU University Hospital, LMU Munich, Comprehensive Pneumology Center, Member of the German Center for Lung Research (DZL), 81377 Munich, Germany; juergen.behr@med.uni-muenchen.de
* Correspondence: susanne.naehrig@med.uni-muenchen.de

Supplementary Materials

Table S1. Number of pwCF with data for each time point available.

Time point	All pwCF[1]	Full dose	Reduced dose	Male	Female	ppFEV1	Missing values for ppFEV1	BMI	Missing values for BMI
**1**[2]	155	127	28	91	64	151	4	155	
**2**	143	114	29	85	58	142	1	143	
**3**	127	87	40	78	49	124	3	126	1
**4**	111	71	40	68	43	108	3	111	
**5**	95	54	41	58	37	93	2	95	
**6**	76	40[3]	36	44	32	76		76	
**7**	49	23	26	29	20	47	2	49	
**8**	25	12	13	16	9	24	1	25	
**9**	5	3	2	4	1	5		5	

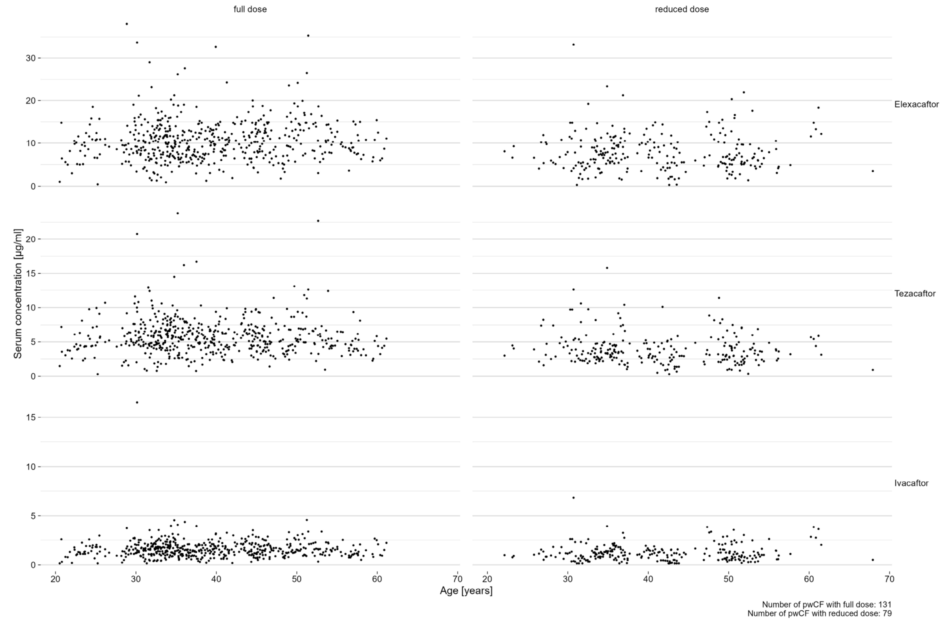

Figure S1. Effects between age and serum concentrations of ETI.

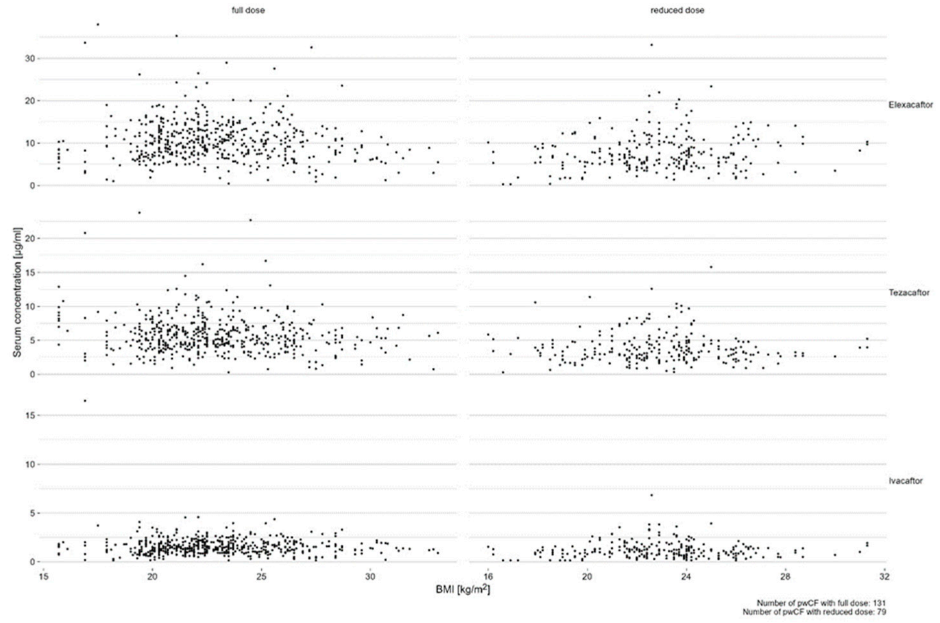

Figure S2. Effects between BMI and serum levels of ETI.

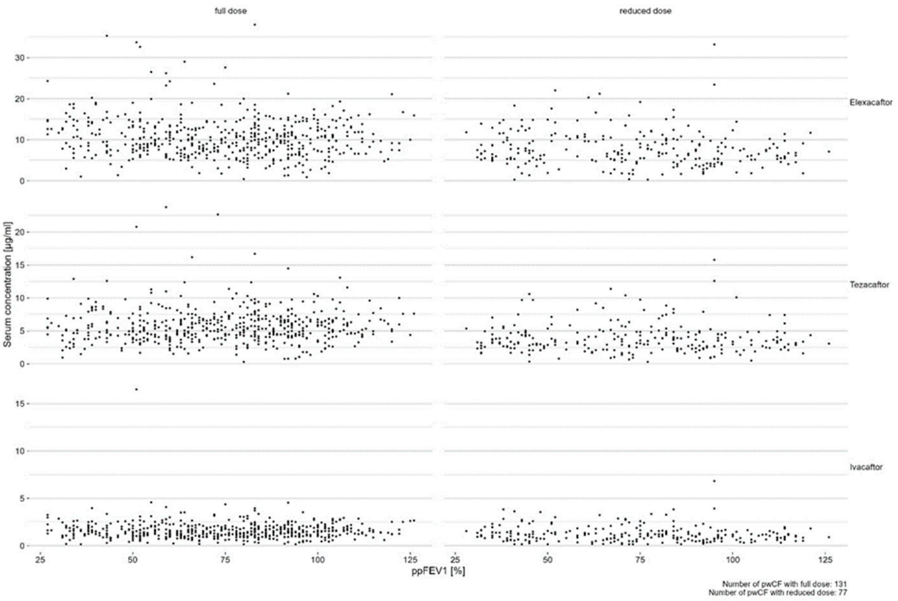

Figure S3. Effects between ppFEV1 and serum levels.

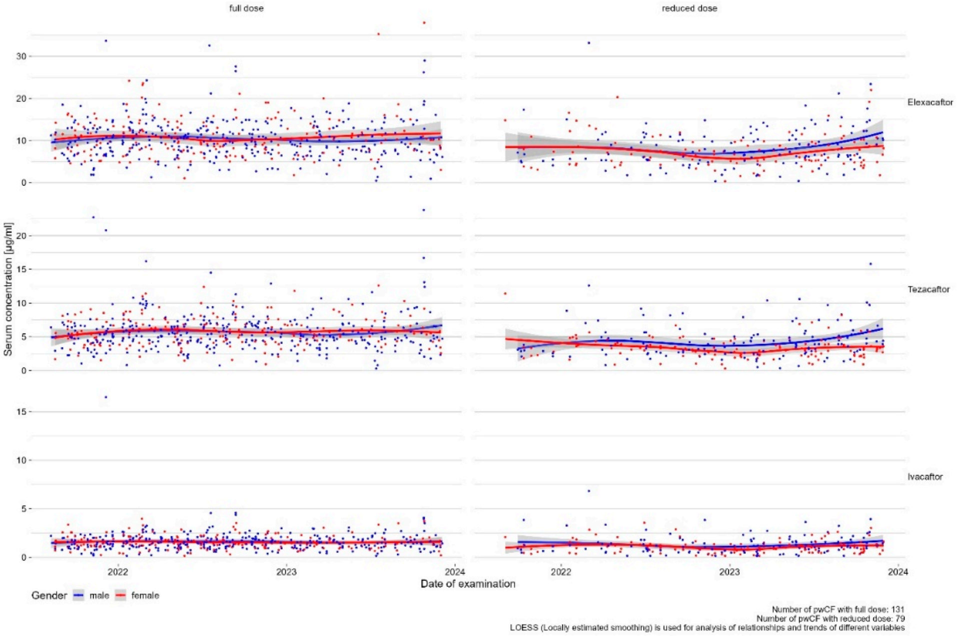

Figure S4. Effects between gender and serum levels.
